# A Mechanical Sensor Using Hybridized Metamolecules

**DOI:** 10.3390/ma12030466

**Published:** 2019-02-03

**Authors:** Haohua Li, Xiaobo Wang, Tian Yang, Ji Zhou

**Affiliations:** State Key Laboratory of New Ceramics and Fine Processing, School of Materials Science and Engineering, Tsinghua University, Beijing 100084, China; lhh14@mails.tsinghua.edu.cn (H.L.); wangxb14@mails.tsinghua.edu.cn (X.W.); yangt16@mails.tsinghua.edu.cn (T.Y.)

**Keywords:** hybridization induced transparency (HIT), metamolecule, collective mode, dielectric, elastic layer, tunable, coupling strength, sensing

## Abstract

Hybridized metamaterials with collective mode resonance are usually applied as sensors. In this paper, we make use of one Mie-based hybridized metamolecule comprising of dielectric meta-atoms and an elastic bonding layer in order to detect the distances and applied forces. The hybridization induced splitting results in two new collective resonance modes, of which the red-shifted mode behaves as the in-phase oscillation of two meta-atoms. Owing to the synergy of the oscillation, the in-phase resonance appears as a deep dip with a relatively high Q-factor and figure of merit (FoM). By exerting an external force, namely by adjusting the thickness of the bonding layer, the coupling strength of the metamolecule is changed. As the coupling strength increases, the first collective mode dip red-shifts increasingly toward lower frequencies. By fitting the relationship of the distance–frequency shift and the force–frequency shift, the metamolecule can be used as a sensor to characterize tiny displacement and a relatively wide range of applied force in civil engineering and biological engineering.

## 1. Introduction

Coupling between meta-atoms causes a hybridization effect in metamaterials [1,2,3], which usually results from asymmetric elements in metamaterials, and results in energy level splitting [4,5,6,7,8,9,10]. Because of the sharp resonance dips and sensitivity to external factors, the frequency shift of such hybridized resonances can be used to characterize various external information [11,12,13], like the refractive index, and can thus be applied in biological and other engineering [14,15,16,17]. In addition, small displacements between the coupled meta-atoms lead to frequency shifts of the collective modes, which can be utilized for mechanical sensing based on electromagnetic spectra. The interplays between mechanics and electromagnetics have already been applied for advanced sensing, super-resolution imaging, and non-destructive detection. These functions are realized by specially designed artificial structures, like phoxonic crystal cavity [18], piezoelectriclike meta-atoms [19], and cavity-assisted optical lattice clock [20]. Such kinds of multi-physics metastructures have inspired our design, which involves both mechanical and electromagnetic factors. By combining coupling-sensitive metamaterials with flexible materials, tiny displacement and applied forces can be detected by frequency shifts. Pryce proposed one structure fabricated on an elastic substrate, of which the reflectance spectra can be shifted by stretching the substrate [21]. Zheng obtained one strain-sensitive flexible metamaterial by varying the asymmetry of the electric-magnetic coupling structure [22]. It can be inferred that by using an adjustable soft material to bond the meta-atoms together, the hybridized collective mode of the metamolecule can be actively tuned through external forces.

However, most previous research about such hybridization effects focuses on visible or infrared wavelengths [23,24,25,26,27]. Here, we take advantage of a Mie-resonance of a dielectric in a microwave range [28,29,30,31,32] to realize it. By combining the meta-atoms with an elastic silica gel layer, the distance between the two dielectric meta-atoms can be adjusted regularly, and the transmission spectrum shifts accordingly. Based on this, we can make use of a frequency shift to characterize the displacement and the external applied force. By placing two dielectric meta-atoms at certain observation points, the movement of the two points can be fitted via a frequency shift of the metamolecule; moreover, because of the natural properties of the bonding elastic layer, the amplitude of the applied force can also be calculated by a frequency shift of metamolecule. Compared with the higher frequency domain, a microwave is easier to obtain and has weaker radiation. Besides, the materials used in our experiment are all biocompatible, which is promising for application in vivo.

Some research has also successfully taken advantage of the coupling phenomenon of Mie-resonance in dielectric to characterize the strain and applied force [33]. However, compared with a single composite “homonuclear diatomic molecule”, a metamolecule composed of two different meta-atoms has a well-performing combination of Q-factor and figure of merit (FoM) to external factors, like tiny displacement. The two indexes are vital for sensing, and they benefit from the synergy effect of the first hybridized collective mode.

## 2. Materials and Simulation

**Figure 1:**
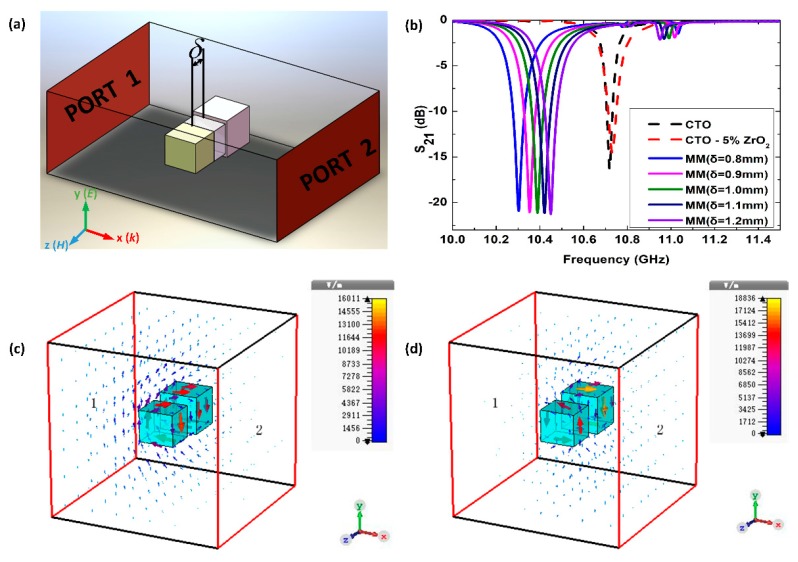
(**a**) Schematic diagram of a metamolecule composed of two dielectric cuboids bonded by a silica gel layer with a thickness *δ* in a waveguide. (**b**) Simulated transmission curves of CaTiO_3_ (CTO) and CaTiO_3_ with 5% ZrO_2_ (CTO–5%ZrO_2_), meta-atoms (dash lines) and metamolecule (abbreviated as MM) of different spacings *δ* (solid lines). At (**c**) 10.45 GHz and (**d**) 10.95 GHz, the displacement electric fields are distributed in two collective modes of metamolecules with *δ* = 1.2 mm.

Figure 1a depicts the schematic configuration of the adjustable metamolecule. The dielectric meta-atoms are bonded by a silica gel layer, which is soft enough to be compressed by an external force. Meta-atoms are made of pure CaTiO_3_ (CTO) and CaTiO_3_ with 5% ZrO_2_ (CTO–5%ZrO_2_) cutting into cuboids of 2 × 1.8 × 1.8 mm^3^ and 2.2 × 2.2 × 2 mm^3^ in size, respectively. The complex relative permittivity of pure CTO and CTO–5%ZrO_2_ are Ɛ_r_ = 160, tan*δ* = 0.001 and Ɛ_r_ = 119, tan*δ* = 0.002, respectively. The relatively high real part and low imaginary part of the permittivity results in a strong resonance amplitude and high Q-factor.

We performed a simulation using the commercial software package CST (Computer Simulation Technology) microwave studio to calculate the transmission spectra of both the single meta-atoms and the assembled metamolecule. As Figure 1a shows, the incident plane wave propagates along the x-axis, with the magnetic field acting along the z-axis and the electric field along the y-axis. The dash lines in Figure 1b show the transmission spectra of single meta-atoms. When we only put a single meta-atom in the center of the waveguide, the two meta-atoms produced dramatic transmission dips at very similar frequencies of ca. 10.72 GHz.

To simulate the transmission behavior of the metamolecule, we set the spacing *δ* between the two meta-atoms (i.e., the thickness of the elastic layer) as 1.2 mm; the value is smaller than the size of the working wavelength, and it is expected that the coupling effect between the two meta-atoms is strong enough to affect the resonance of the metamolecule. The solid lines in Figure 1b show the transmission response S_21_ of the metamolecule. One noticeable transparent window is supplanting the initial dip of the meta-atoms; furthermore, two significant dips are found to appear. The coupling within the metamolecule results in the transmission dip of the meta-atoms splitting, and induces a hybridization transparency.

When the spacing *δ* decreases from 1.2 to 0.8 mm with a step of 0.1 mm, the transparent window widens. The first and second collective modes of metamolecule accelerate to a lower and higher frequency, as shown in Figure 1b. The transparency occurs because of the strong coupling between the two meta-atoms and the formation of new collective resonance dips. Two meta-atoms with the coincident first-order Mie resonance frequency can be regarded as two oscillators. Their combination is characterized by simultaneous “two-particle model” equations [34]. As the effective susceptibility χ approaches infinity, the two edge frequencies that define the stopband of our metamolecule are obtained as follows:

(1) $$\begin{array}{l} \omega_{-}=\sqrt{\omega_{0}{}^{2}-\kappa^{2}}\\ \omega_{+}=\sqrt{\omega_{0}{}^{2}+\kappa^{2}} \end{array}$$

where *ω*_0_ is the first-order Mie resonance frequency of the two meta-atoms, and *κ* represents the coupling strength between the meta-atoms. Thus, the width of the transparent window can be calculated as follows:

(2) $$\Delta \omega =\omega_{+}-\omega_{-}\approx \frac{\kappa^{2}}{\omega_{0}}\propto \kappa \propto \delta$$

where the coupling strength *κ* is positively correlated to the coupling distance *δ*. By exerting an external force to adjust *δ* between two meta-atoms, we can create an actively tunable hybridization induced transparent window of metamolecules. When the transparent window becomes wider, the in-phase collective mode of the metamolecule red-shifts to a lower frequency, while the out-of-phase collective mode blue-shifts to a higher frequency.

As Figure 1b shows, the resonance dip of the first collective mode is much stronger than that for either of the individual meta-atoms, while the dip of the second collective mode is much weaker. This can be explained by the mechanism of the dielectric “diatomic molecules” hybridization model. In our previous work [35], we explained the hybridization phenomenon of Mie-based metamolecule by observing the distribution of the displacement electric current at both collective resonance frequencies. The first order Mie resonance behaves as a magnetic resonance, formed by the circular arrangement of displacement electric currents inside a dielectric [36]. For the in-phase mode, magnetic dipoles of both meta-atoms oscillate in the parallel direction, and thus strengthen the response by inducing the magnetic fields of the same direction, as Figure 1c shows. While for the out-of-phase mode, the displacement electric current of the two meta-atoms oscillate in anti-parallel directions, and the response is weakened by the counteracting effect of the anti-direction oscillation, as Figure 1d shows.

**Figure 2:**
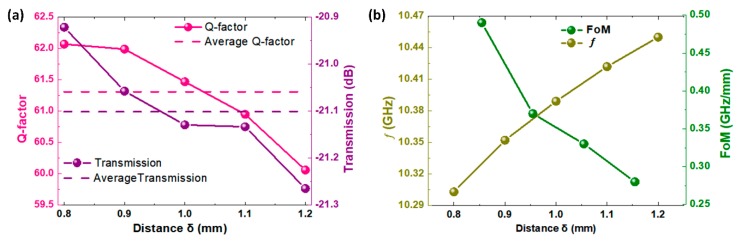
(**a**) Calculated Q-factors (pink dots) and simulated transmission amplitude (purple dots) of metamolecules with distances of δ = 0.8 mm, 0.9 mm, 1.0 mm, 1.1 mm and 1.2 mm. Calculated average Q-factors (pink dash lines) and transmission amplitude (purple dash lines) with the above distances δ. (**b**) Simulated resonance frequencies ƒ (dark yellow dots) and calculated figure of merit (FoM) (green dots).

The first collective mode is formed by the synergy of two in-phase magnetic oscillations, and appears as a stronger resonance dip compared with either of the two meta-atoms. The dip shifts as the coupling strength changes, thus it can be taken advantage of to reflect the coupling displacement. Considering that Q-factor and figure of merit (FoM) are two vital indexes of sensing devices. We calculated the Q-factors of the resonance dips of different spacing *δ* from 1.2 mm to 0.8 mm, as Figure 2a shows. The Q-factor can be expressed as follows:

(3) $$Q=\frac{f}{FWHM}$$

where FWHM represents the full-width at half maximum. Using a metamolecule with *δ* = 0.8, 0.9, 1.0, 1.1, and 1.2 mm as examples, the average Q-factor of the metamolecule is 61.31, and average transmission amplitude is −21.1 dB. Sensors with a high Q-factor and strong resonance amplitude are expected to have an outstanding performance in detection fields, owing to their sharp and deep dips.

Furthermore, as Figure 1b shows, the frequency shifting rates change with spacing δ. We put forward a concept called figure of merit (FoM) to characterize it, as follows:

(4) $$FOM=\frac{f-f^{\prime }}{\delta -\delta^{\prime }}$$

where f and $f^{\prime }$ represent for the frequency of the first hybridized resonance mode when the spacing is δ and $\delta^{\prime }.$ This parameter illustrates the sensitivity to coupling spacing of the metamolecule. We calculated the FoM for the different δ of this metamolecule, and the results are shown in Figure 2b. It can be seen that when the spacing is reduced, the dip red-shifts at increasing rates. This is because the collective mode is a response to the interaction effect, and this effect becomes even more obvious for coupled meta-atoms with smaller spacing. It can be used to measure spatially distributed information over very small distances.

As both Q-factor and FoM should be taken account of for sensing, we make a comparison of these two indexes among metamolecules and their constituent “homonuclear diatomic molecule”. As shown in Table 1 and Table 2, our metamolecule makes a good trade-off between Q-factor and FoM, compared with (CTO–5%ZrO_2_)–(CTO–5%ZrO_2_) and CTO–CTO “homonuclear diatomic molecule”. Owing to the sharpness and sensitivity of the in-phase resonance dip of the metamolecule, it is suitable to be used as a sensor to characterize distance and mechanical information.

## 3. Results and Discussion

**Figure 3:**
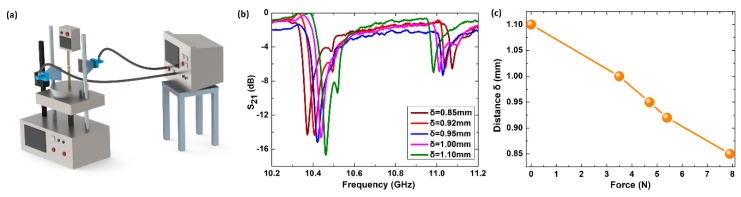
(**a**) Schematic diagram for mechanic-microwave measurement setup. (**b**) Measured transmission curves of metamolecule with various distances *δ*. (**c**) Measured distances *δ* under different applied forces F.

Assembling the metamolecule with an elastic layer ensures that the spacing between the coupled meta-atoms can be adjusted actively and freely. Silica gel is used to bond two dielectric meta-atoms to form a metamolecule. Considering the natural mechanical properties of the silica gel, not only the distance, but also the applied force can be characterized indirectly by a frequency shift. An experiment setup was established to observe the transmission spectra of the metamolecule under different forces, as shown in Figure 3a. Two XB-WA-90-N horn antennas connected to an Agilent N5230C vector network analyzer (Santa Rosa, CA, USA) was used as microwave emitter and receiver. A dynamometer was utilized to exert and measure forces on our metamolecule sample, with two wooden sticks touching the metamolecule and exerting the force. Meanwhile, the dynamometer displayed the compressed dimension of the metamolecule and the magnitude of force applied on the metamolecule. We measured the transmission spectra of the metamolecule with various thickness *δ* from 1.10 mm to 0.85 mm, as shown in Figure 3b.

When there is no external force, the thickness *δ* of the silica gel is 1.10 mm, and the transmission dip of the first collective mode is found at 10.463 GHz, agreeing well with simulation results in Figure 1b. When external forces of 3.5 N, 4.7 N, 5.4 N, and 7.9 N are applied by dynamometer gradually, *δ* are read to be 1.00 mm, 0.95 mm, 0.92 mm, and 0.85 mm, respectively, by scale meter after stabilization, as shown in Figure 3c. The transmission dips of the in-phase mode, meanwhile, are measured to have become 10.438 GHz, 10.422 GHz, 10.406 GHz, and 10.372 GHz using a vector network analyzer, respectively; these results can be seen in Figure 3b. After a loading force of more than 7.9 N, the thickness of the silica gel almost cannot be compressed easily because of the natural hardening effect of the material itself. Thus, we study the behavior of the metamolecule when the applied force, F, ranges from 0 N to 7.9 N, and spacing *δ* varies from 1.10 mm to 0.85 mm.

**Figure 4:**
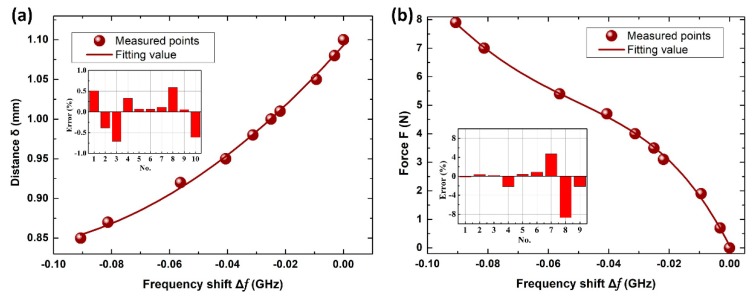
(**a**) Measured distances *δ* of different frequency shifts Δƒ (wine red points) and the fitting relationship between *δ* and Δƒ (wine red curve). (**b**) Measured applied forces, F, of different frequency shifts, Δƒ, (wine red points) and the fitting relationship between F and Δƒ (wine red curve). The insets in Figure 4a,b represent for the errors of the fitting values and the measured values of various distances, *δ*, and applied forces, F.

From the measured results above, we found that the resonance frequency shift can be taken advantage of to reflect the external applied force and the distance between the two meta-atoms. To make the metamolecule a sensor for general applications, we fitted the relationship between the frequency shift (Δƒ) and the applied force (F), as well as Δƒ and distance *δ*, using the Origin Software package. We also tested five other data points, which are shown in Figure 4a,b.

As mentioned in Figure 2b, the smaller *δ* is, the faster a transmission dip red-shifts. We therefore used a high-order equation to fit the non-linear law of *δ* to Δƒ. However, it should be noted that too great an order of a polynomial increases the computational workload, which limits the applicability and efficiency of such a method. With considerations to both accuracy and practicality, we have fitted the relationship between Δƒ and *δ* using a quadratic Equation (5), as follows:

(5) $$\delta =1.09+4.11\times \Delta f+16.27\times \Delta f^{2}$$

We calculated the errors between our fitted results and the measured results to see how accurately this equation can predict the tiny displacement from the frequency shift. From the inset of Figure 4a, it can be seen that the equation fits the experimental results well. The errors of the fitting values and measured values are less than 1.0%.

With regard to the relationship between Δƒ and F, we need to consider both the relationship of *δ*–Δƒ and F–*δ*; the former has already been discussed as being quadratic. For F–*δ*, because silica gel is a soft material, *δ* for the metamolecule changes in a non-linear way as a function of F. If the relationship between *δ* and Δƒ is considered to be quadratic in nature, then F–Δƒ should be biquadratic, the following equation can be obtained by fitting:

(6) $$F=0.069-2.08\times 10^{2}\times \Delta f-3.51\times 10^{3}\times \Delta f^{2}-3.12\times 10^{4}\times \Delta f^{3}-8.07\times 10^{4}\times \Delta f^{4}$$

As Figure 4b shows, this biquadratic equation fits well with the results from the experiment, and the errors between the fitting and measured values are found to be less than 8%.

It is worth mentioning that the applied forces can cover the range from 0 N to 7.9 N, signifying that a tiny applied force can also be detected. When the force increases, because of the hardening effect of the silica gel itself, the thickness cannot be compressed and the spacing is hard to change. So, we studied the displacement mainly in the range from 0.8 mm to 1.2 mm. By changing the thickness or type of the soft bonding materials, we can obtain a sensor of even smaller detecting distances.

## 4. Conclusions

Based on the discussion above, by combining two meta-atoms together, we have designed a hybridized metamolecule. Considering the trade-off between Q-factor and FoM, it is more outstanding to characterize the distances and applied forces than single component “homonuclear diatomic molecule”. By bonding two meta-atoms with an elastic material layer, we can actively tune the distances between two coupled meta-atoms via external forces, thus leading to transmission spectra shifting. By fitting the relationship of the resonance frequency shifts with distances and applied forces, we obtained a wireless and telemetric sensor to detect forces and distances for more general use.

Such devices have obvious advantages for detecting tiny displacements and forces. By altering the thickness or type of soft material, we can get an even wider detection range, as needed. Meanwhile, considering the working frequency and fabrication processing, it has advantages of low radiation, is simple-equipped, and is low cost. In addition, both dielectrics and silica gels are biocompatible, which makes the metamolecule promising for sensing in vivo. Our findings have great potential for the detection of tiny displacements and applied forces in civil engineering and biological fields.

## Figures and Tables

**Table 1:** Q-factors of CaTiO_3_ (CTO) “homonuclear diatomic molecule”, CaTiO_3_ with 5% ZrO_2_ (CTO–5%ZrO_2_) “homonuclear diatomic molecule”, and metamolecules with various distances δ and their average values of these distances.

Q-Factors	δ					
Diatomic Molecule	0.8	0.9	1.0	1.1	1.2	Average
CTO–CTO	76.50	75.69	75.40	74.55	73.70	75.168
(CTO–5%ZrO_2_)–(CTO–5%ZrO_2_)	50.46	49.56	49.26	48.72	48.18	49.236
Metamolecule	62.07	61.99	61.47	60.95	60.06	61.308

**Table 2:** Figure of merit (FoM) of CTO “homonuclear diatomic molecule”, CTO–5%ZrO_2_ “homonuclear diatomic molecule”, and metamolecule with various distances δ and their average values of these distances.

FoM	δ					
Diatomic Molecule	0.8	0.9	1.0	1.1	1.2	Average
CTO–CTO	0.42	0.36	0.32	0.28	\	0.345
(CTO–5%ZrO_2_)–(CTO–5%ZrO_2_)	0.64	0.37	0.32	0.30	\	0.4075
Metamolecule	0.49	0.37	0.33	0.28	\	0.3675
